# Hybrid Fixation Technique for Femoral Stem Revision in B3 Periprosthetic Fractures: A Report of Two Cases

**DOI:** 10.1016/j.artd.2025.101645

**Published:** 2025-02-28

**Authors:** Moein Akbari Javar, Sina Esmaeili, Shahram Shokraneh, Mohammad Ghorbanzadeh, Seyed Mohammad Javad Mortazavi

**Affiliations:** a Joint Reconstruction Research Center, Tehran University of Medical Sciences, Tehran, Iran; b Department of Orthopedic Surgery, Imam Khomeini Hospital Complex, Tehran University of Medical Sciences, Tehran, Iran

**Keywords:** Periprosthetic femoral fractures, Vancouver type B3, Cementless prosthesis, Case report

## Abstract

Periprosthetic femoral fractures (PPFs) are challenging complications following total hip arthroplasty, especially in cases with compromised bone stock. We present 2 cases of Vancouver Type B3 PPF treated with a monoblock cementless long-stem prosthesis, using cement only for distal fixation. This technique provided initial stability, allowing early mobilization and eventual fracture healing with bony ingrowth. Radiographic follow-up demonstrated successful outcomes with no significant complications. Our results support the potential of this method for treating complex PPF cases, offering a solution for patients with inadequate bone quality where standard cementless fixation may be insufficient.

## Introduction

Periprosthetic femoral fracture (PPF) is a serious complication following total hip arthroplasty (THA) [1]. With an aging population and the success of arthroplasty procedures, the number of joint replacements has increased significantly over the past decade. As THA becomes more common and life expectancy rises, the incidence of PPF has also grown [2]. The reported incidence ranges from approximately 1% after primary THA to 4% after revision THA [[3], [4], [5]]. This trend is expected to continue, with projections suggesting an increasing rate of THA procedures and a corresponding rise in PPF cases through 2030 [6].

Several classification systems have been proposed for PPFs, with the Vancouver classification by Duncan et al. being the most widely accepted one [7,8]. This system categorizes fractures based on their location, the stability of the femoral component, and the quality of the remaining bone stock [7,9,10]. In cases where the prosthesis is loose, and there is deficient proximal bone stock in the femur (Vancouver Type B3 fractures), revision of the femoral component and prosthesis exchange is recommended. This approach addresses both the instability of the prosthesis and the fracture itself by providing intramedullary support through the use of long-stemmed implants commonly employed in revision procedures [11,12]. During revision, the prosthesis can be replaced with either a cemented or non-cemented stemmed implant. However, cemented stems carry the risk of cement forced into the fracture site, potentially impeding union and healing. In addition, the effectiveness of modular, non-cemented stems in Vancouver Type B3 fractures, particularly when used with extended trochanteric osteotomies, remains uncertain, especially when the distal femur has poor bone quality [8,11].

To the best of our knowledge, this study is the first to treat 22 patients with PPFs and poor bone stock using a monoblock cementless long-stem prosthesis, with cement fixation applied only to the distal portion of the stem. We describe the technique, report the outcomes, and provide the clinical follow-up for both cases.

## Case histories

### Case 1

A 70-year-old woman presented to the emergency department with a 3-day history of right groin pain. Her medical history was significant for multiple myeloma (MM) and a right femoral neck fracture treated 1 month prior with a cemented bipolar hemiarthroplasty using a CORAIL stem (Depuy Synthes, Raynham, MA). Following hemiarthroplasty, the patient commenced accelerated weight-bearing with a walker. In addition, she had undergone a left THA for hip osteoarthritis approximately 10 years earlier.

**Figure 1:**
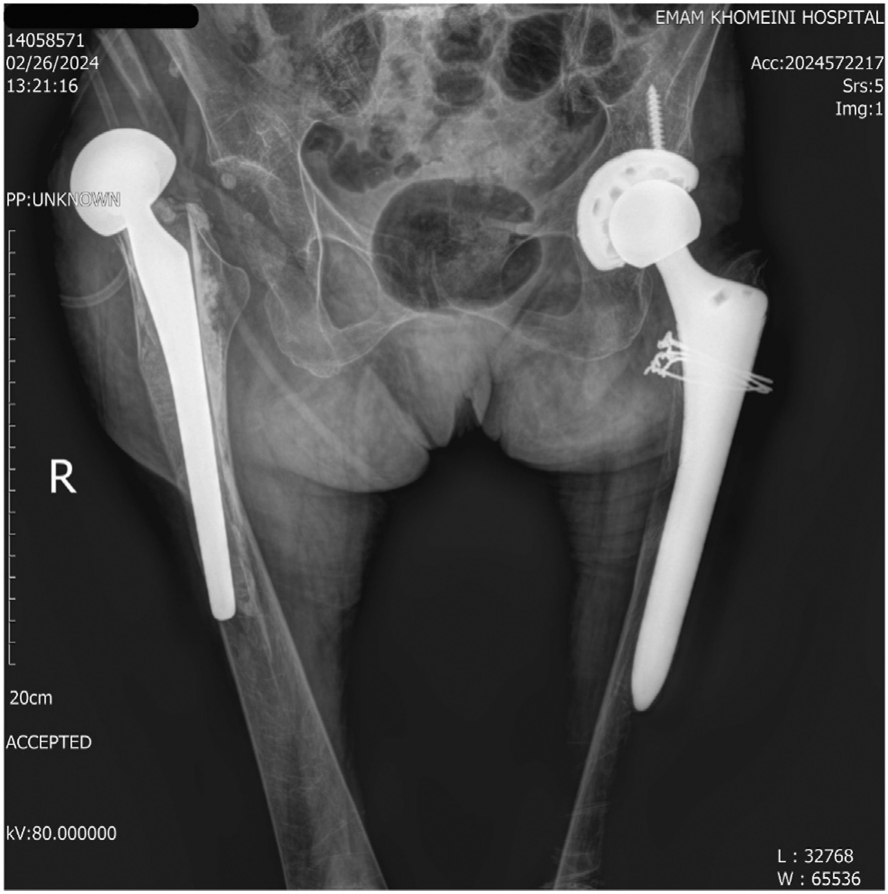
Preoperative radiograph of case 1.

Upon clinical examination, the patient exhibited tenderness in the right hip region. In addition, inflammatory markers, including erythrocyte sedimentation rate and C-reactive protein, were evaluated to exclude the possibility of infection. Radiographic imaging, including anteroposterior and lateral views of the pelvis, revealed a PPF, classified as Vancouver type B3 (Fig. 1). Given these findings, a decision was made to perform revision arthroplasty.

#### Surgical procedure

**Figure 2:**
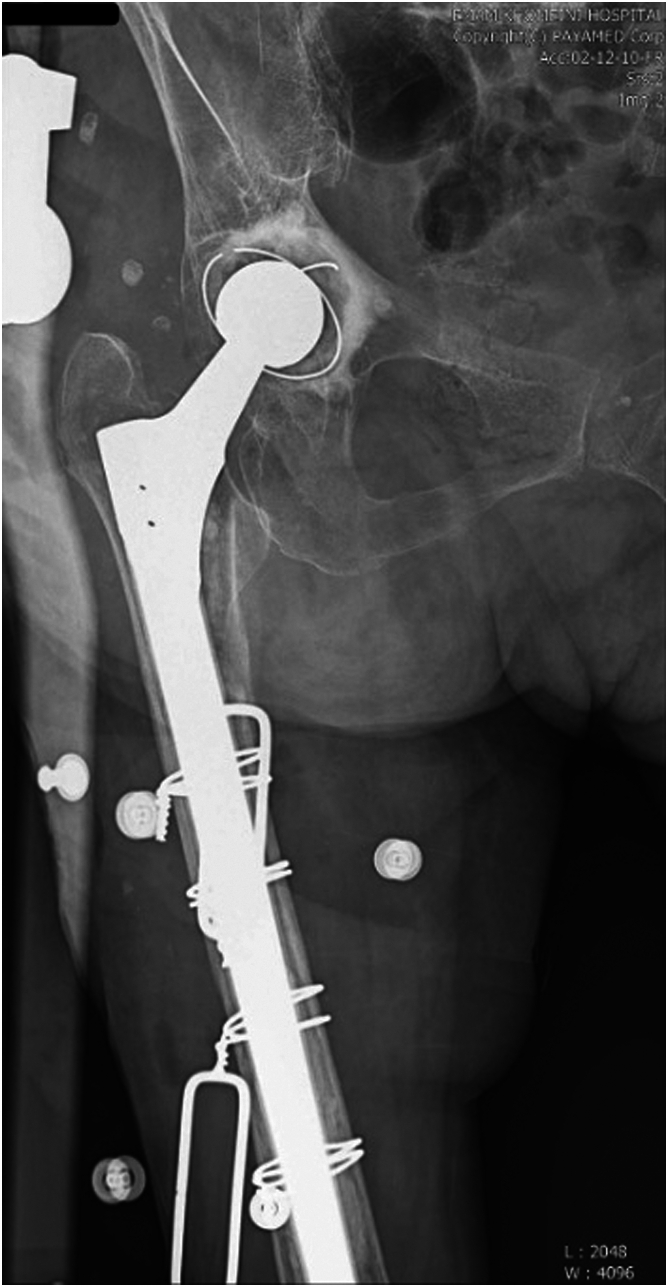
Postoperative radiograph of case 1.

Revision surgery was conducted under general anesthesia with the patient positioned laterally. A lateral approach was used to access the hip. Intraoperatively, acetabular damage was identified, necessitating reaming of the acetabular fossa. A 47-mm cemented ZCA All-Poly acetabular cup (Zimmer, Warsaw, Indiana) was implanted. The cement mantle and femoral stem were removed through the PPF site. To prevent fracture propagation during reaming and stem impaction, a prophylactic cerclage wire was placed 1 cm distal to the PPF site. The distal femur was reamed with sized tapered reamers until the cancellous bone at the proximal entry of the distal segment, visible under direct visualization, was adequately removed, leaving the cortical bone intact. Due to the quality of bone, large diameter of the canal, and the absence of a converging cortex, long cementless stems designed to fit the diaphysis did not achieve enough scratch fit within the distal canal. To address this, we decided to use cement for the fixation of the distal part of the stem. To ensure that cement did not enter the fracture site, the stem was cemented to the distal segment. Any excess cement was meticulously removed before proceeding further. After achieving joint reduction, the remaining fracture fragments were positioned around the stem. To achieve a proper cementing technique, the distal femoral canal was closed beyond the length of the stem using absorbable gelatin sponge (Gelfoam, Pfizer, New York, NY) wrapped in Surgicel (Original, Ethicon Inc, Bridgewater, NJ). Pressurized cement was then introduced into the distal canal, providing a foundation for stable stem fixation. A diaphyseal fitting long cementless stem (Wagner SL, Zimmer; 265 mm in length, 17 mm in diameter, with a 28-mm head) was inserted in the distal part. After the joint reduction, the proximal fracture fragments were reduced around the distally fixed stem and stabilized with 2 wires. The objective of this approach was to provide initial stability through cement fixation, allowing for eventual fracture union and osteointegration of the proximal segment around the cementless stem (Fig. 2).

The wound was closed in layers, and dressing was applied. Postoperatively, the patient was mobilized on the first day, with weight-bearing as tolerated. She was discharged on postoperative day 3 and prescribed 80 mg of aspirin every 12 hours for 1 month as thromboprophylaxis.

#### Follow-up

Postoperative radiographs were obtained on the day of surgery, then at 4 ,8, 12, and 24 weeks postoperatively. No evidence of bone resorption or heterotopic ossification was noted. The patient showed accelerated weight-bearing without extension of the PPF or development of new fractures. Final radiographs showed successful bony ingrowth in the proximal part of the stem (Fig. 2). The last follow-up visit was conducted remotely at 11 months postoperatively, as the patient was unable to return for an in-person visit due to the distance. During this follow-up, the patient reported no complaints of complications, pain, or difficulty with ambulation.

### Case 2

**Figure 3:**
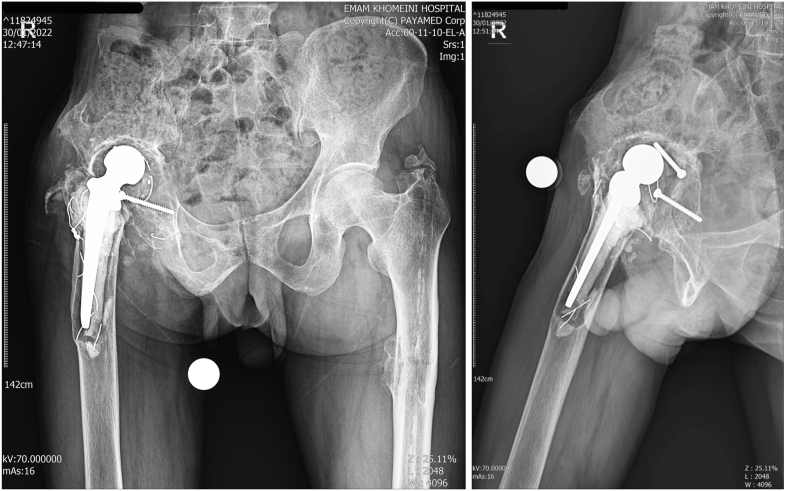
Preoperative radiograph of case 2.

A 65-year-old male presented with complaints of right hip pain and limping. The patient had a history of pelvic osteotomy 45 years prior for Perthes disease, followed by a THA 28 years ago. Clinical examination revealed a 4-cm shortening of the right lower extremity. Levels of erythrocyte sedimentation rate and C-reactive protein were assessed to rule out infection. Radiographs showed a loose acetabular cup (Paprosky type 2a), a loose femoral stem (Paprosky type 3a), and a PPF classified as type B3 according to the Vancouver classification (Fig. 3).

#### Surgical procedure

**Figure 4:**
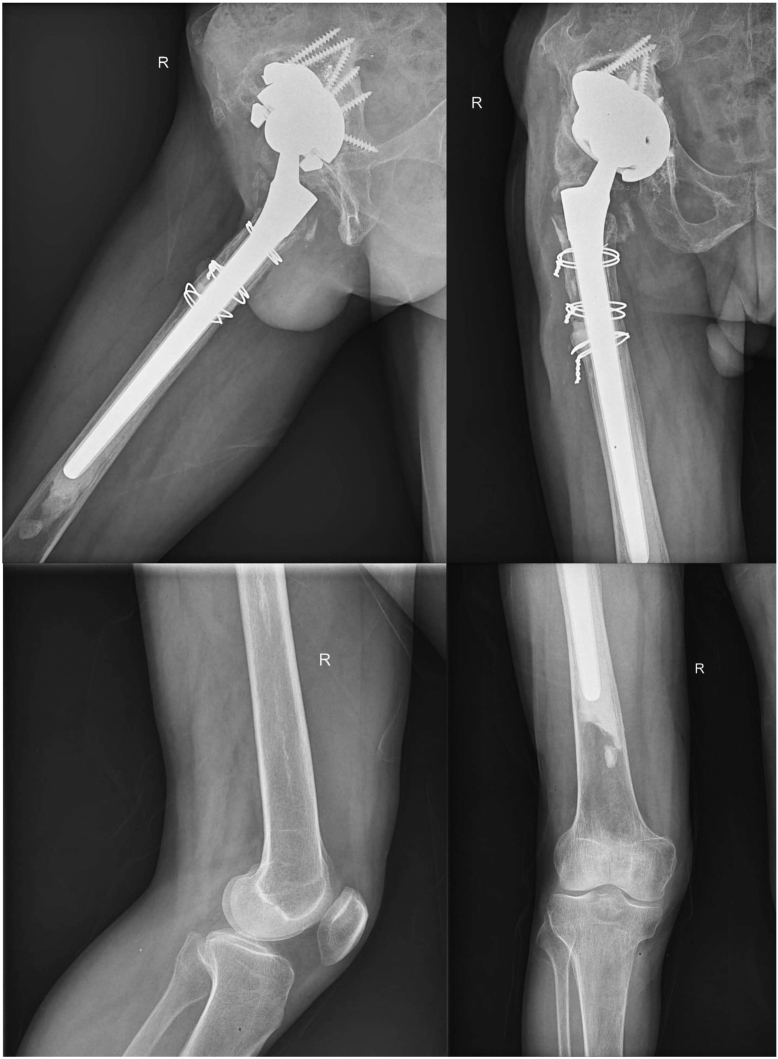
Final follow-up radiograph of case 2.

A revision THA was planned and performed under general anesthesia with the patient in the lateral position, utilizing a lateral approach. The first step involved removal of the polyethylene acetabular liner. Bone grafting was carried out at the acetabular site, after which a 25 × 54-mm highly porous tantalum augment (Trabecular Metal, Zimmer Biomet, Warsaw, IN) was impacted into place. A 64-mm acetabular component with a highly porous trabecular tantalum fixation surface (Continuum Acetabular System, Zimmer Inc, Warsaw, IN) was then screwed into the proper position. Attention was then directed to the femoral component. The femoral stem and cement mantle were removed through the PPF site. To prevent further fracture propagation during reaming, a prophylactic cerclage wire was placed 1 cm distal to the most inferior point of the PPF. The distal femur was reamed using tapered reamers. Due to the compromised isthmus, dilated femoral canal, and the absence of a convergent cortex, a long cementless stem designed for diaphyseal fixation did not achieve a complete scratch fit. To address this, the distal canal was blocked 2 cm longer than the stem length using an absorbable gelatin sponge (Gelfoam) wrapped in Surgicel. Cement was then pressurized into the distal canal, and a long cementless stem (265 mm in length, 20 mm in diameter) was inserted. Following joint reduction, the proximal fracture fragments were aligned and secured with cables around the femoral stem. The greater trochanter and abductor muscles were repaired using a fiber wire. Finally, the wound was closed in layers (Fig. 4).

Postoperatively, partial weight-bearing was allowed on the first postoperative day.

#### Complications and management

Three months after the surgery, the patient experienced a hip dislocation. A closed reduction was performed under general anesthesia. Post-reduction radiographs confirmed proper placement of the femoral head within the acetabular shell. Due to persistent abductor weakness, a decision was made to revise the acetabular liner to a 36-mm Trilogy constrained liner (Biomet, Warsaw, IN) to prevent further dislocations.

#### Follow-up

The patient was followed up for 35 months. Radiographs demonstrated successful bony ingrowth in the proximal part of the femoral stem, with no evidence of complications such as loosening, fracture extension, or infection (Fig. 4).

It is important to clarify that the technique utilized in this case report is classified as off-label due to a cementless stem being inserted with a partial cementation. Utilization of this technique is experimental, and further follow-up is necessary to validate this off-label use of the implant.

Written informed consent was obtained from both patients for the publication of this case report.

## Discussion

Periprosthetic fractures following THA pose a substantial challenge to healthcare providers, often requiring urgent and complex management. The literature consistently highlights the elevated risks of complications, reoperations, and increased early mortality rates following PPF, which are associated with poor clinical outcomes and a significant reduction in patients' quality of life. The primary goals of treating PPF are to ensure joint stability, promote fracture healing, and restore the patient’s pre-injury functional level [[13], [14], [15], [16]].

However, certain factors complicate the revision of THA. One key challenge is managing PPF in patients with poor bone quality, which can affect treatment options, surgical approach, and the choice of revision prosthesis. Limited cortical fixation, difficulties in removing cement, and potential stress risers further complicate the process [[17], [18], [19]]. Achieving long-term femoral stem stability is essential to prevent secondary revision and restore previous levels of activity. Nonetheless, in patients with compromised bone quality, securing stable proximal and distal fixation is difficult due to deficient metaphyseal and/or diaphyseal bone stock [20].

Various techniques and implants have been introduced for femoral stem revision, including impaction bone grafting with a cemented polished stem, extensively porous-coated stems, cortical strut allografts, and distal cementless fixation using cortical strut allografts with modular or monoblock femoral stems, or even megaprostheses [17,[20], [21], [22], [23], [24], [25], [26], [27]]. A comprehensive review of these techniques and implants by Ali et al. reported that each approach offers distinct advantages and disadvantages, but none has proven entirely successful in addressing severe bone stock deficiencies [28].

There remains considerable debate regarding the optimal surgical method and implant choice to maximize prosthesis longevity. Some authors recommend that the femoral stem should bypass the bone defect by at least 2 cortical thickness. For long cementless stems, achieving sufficient diaphyseal press-fit fixation requires 4-7 cm of intact isthmus [20,28]. In cases where the isthmus is compromised by fracture comminution or osteoporosis, distal locking or cemented stems are preferred. However, biomechanical and clinical studies have indicated that cemented stems have higher failure rates than cementless implants, making them a less-suitable option for femoral stem revisions [[29], [30], [31], [32]].

In the current technique described, the surgeons aimed to combine the advantages of both cemented and cementless long femoral stems. Cemented stems were avoided due to concerns about bone cement leakage, which could impair fracture healing. Instead, a long monoblock stem was used to bypass the fracture site and bone defects, with cement applied only for distal diaphyseal fixation. This approach allowed for osteogenesis and osteointegration in the proximal regions, resulting in successful fracture union between the proximal and distal segments. At 35 months postoperatively, radiographs showed good fixation of the femoral stems, and the patients were able to walk independently with satisfactory functional outcomes. Further investigation into the long-term outcomes of this technique with a larger cohort is recommended to better confirm its effectiveness and to provide a more comprehensive understanding of potential complications and sustained benefits over time.

## Conclusions

This report highlights a novel approach to treating Vancouver Type B3 PPFs with poor bone stock. Using a monoblock cementless long-stem prosthesis with distal cement fixation proved to be a viable technique in achieving stable fixation, promoting fracture healing, and maintaining successful bony ingrowth. The procedure allowed for early mobilization, minimized complications, and addressed the challenges posed by poor bone quality. Our findings suggest that this method could be a promising option for managing similar cases.

## Conflicts of interest

The authors declare there are no conflicts of interest.

For full disclosure statements refer to https://doi.org/10.1016/j.artd.2025.101645.

## Informed patient consent

The author(s) confirm that written informed consent has been obtained from the involved patient(s) or if appropriate from the parent, guardian, power of attorney of the involved patient(s); and, they have given approval for this information to be published in this case report (series).

## CRediT authorship contribution statement

**Moein Akbari Javar:** Writing – original draft, Project administration, Methodology. **Sina Esmaeili:** Writing – original draft, Methodology. **Shahram Shokraneh:** Writing – original draft, Conceptualization. **Mohammad Ghorbanzadeh:** Writing – review & editing, Writing – original draft. **Seyed Mohammad Javad Mortazavi:** Writing – review & editing, Project administration, Conceptualization.

## Appendix A Supplementary data

Conflict of Interest Statement for Akbari Javar

Conflict of Interest Statement for Esmaeili

Conflict of Interest Statement for Ghorbanzadeh

Conflict of Interest Statement for Javad Mortazavi

Conflict of Interest Statement for Shokraneh
