# Supplementary material for: Hybrid Fixation Technique for Femoral Stem Revision in B3 Periprosthetic Fractures: A Report of Two Cases
Source: Arthroplast Today. 2025 Feb 28;32:101645. doi: 10.1016/j.artd.2025.101645 (PMC11919329; doi:10.1016/j.artd.2025.101645)
Supplement: Conflict of Interest Statement for Javad Mortazavi [file mmc4.docx]

# CONFLICT OF INTEREST STATEMENT

***American Association of Hip and Knee Surgeons***

(Adopted from the American Academy of Orthopaedic Surgeons disclosure statement)

The following form **must be filled out completely and submitted by each author (example, 6 authors, 6 forms).**

**All items require a response. If there is no relevant disclosure for a given item, enter "*None*.”**

**Title: Hybrid Fixation Technique for Femoral Stem Revision in B3 Periprosthetic Fractures: A Report of Two Cases**

1. Royalties from a company or supplier (The following conflicts were disclosed)

**None.**

2. Speakers bureau/paid presentations for a company or supplier (The following conflicts were disclosed)

**None.**

3A. Paid employee for a company or supplier (The following conflicts were disclosed)

**None.**

3B. Paid consultant for a company or supplier (The following conflicts were disclosed)

**None.**

3C. Unpaid consultants for a company or supplier (The following conflicts were disclosed)

**None.**

4. Stock or stock options in a company or supplier (The following conflicts were disclosed)

**None.**

5. Research support from a company or supplier as a Principal Investigator (The following conflicts were disclosed)

**None.**

6. Other financial or material support from a company or supplier (The following conflicts were disclosed)

**None.**

7. Royalties, financial or material support from publishers (The following conflicts were disclosed)

**None.**

8. Medical/Orthopaedic publications editorial/governing board (The following conflicts were disclosed)

**None.**

9. Board member/committee appointments for a society (The following conflicts were disclosed)

**None.**

**Each author must sign AND print or type his/her name, date and submit a separate form**

In addition, one BLINDED Conflict of Interest form (no author names used) should be submitted per manuscript with all author disclosures.


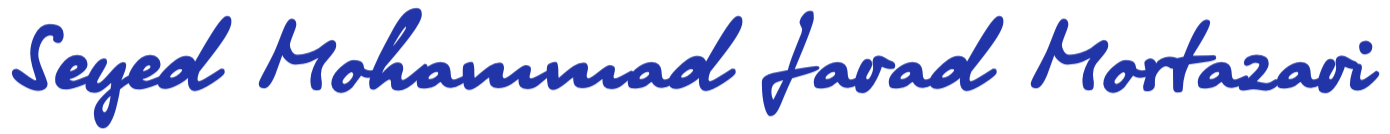
Seyed Mohammad Javad Mortazavi 11/11/2024
